# Supplementary material for: Claustrum neurons projecting to the anterior cingulate restrict engagement during sleep and behavior
Source: Nat Commun. 2024 Jun 26;15:5415. doi: 10.1038/s41467-024-48829-6 (PMC11208603; doi:10.1038/s41467-024-48829-6)
Supplement: Supplementary file 1 — Supplementary Information [file 41467_2024_48829_MOESM1_ESM.pdf]

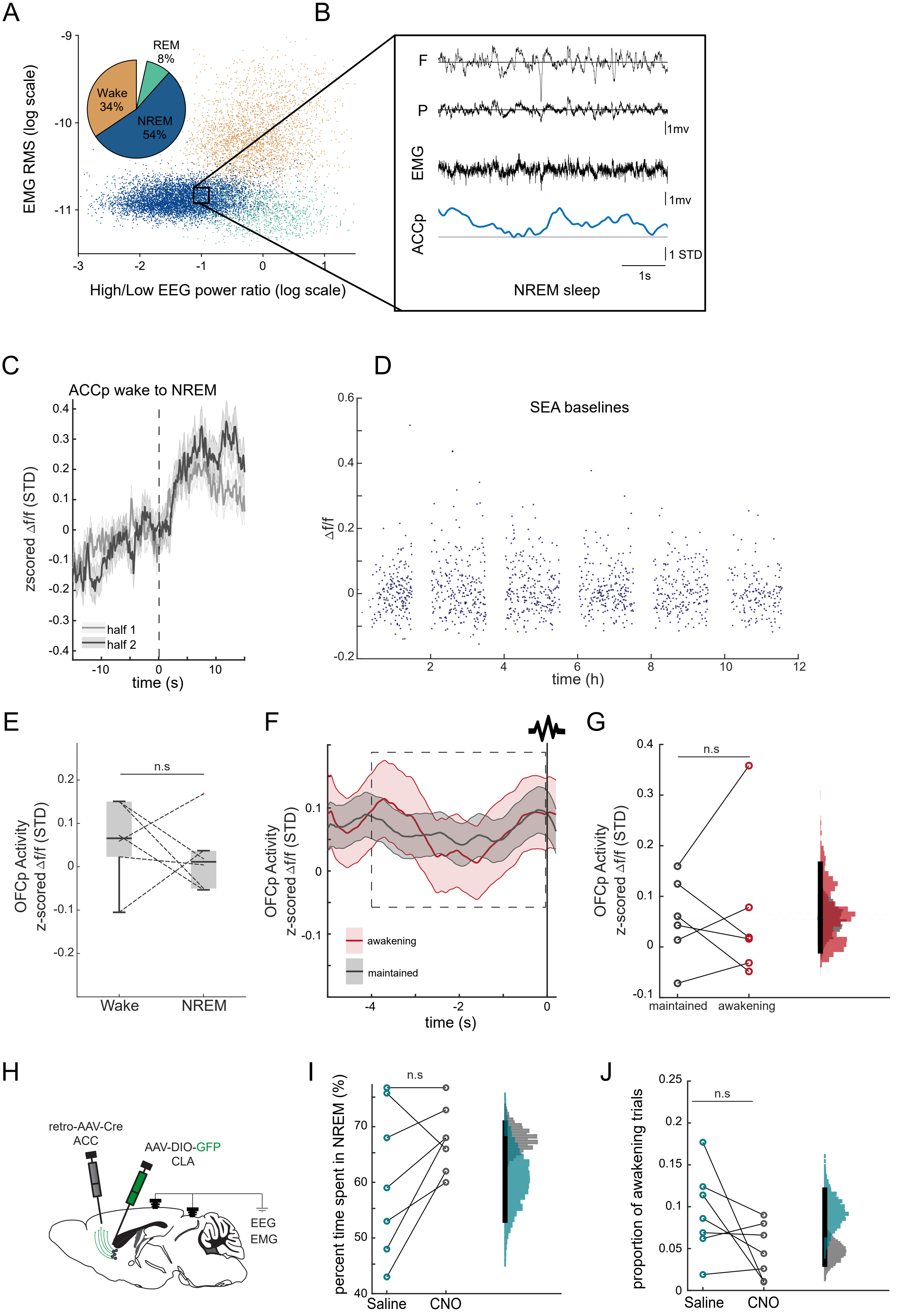

A
B
C
D
E
F
G
H
I
J
EMG RMS (log scale)
High/Low EEG power ratio (log scale)
REM 8%
Wake 34%
NREM 54%
F
P
EMG
ACCp
1mv
1 STD
1s
NREM sleep
ACCp wake to NREM
zscored Δf/f (STD)
time (s)
half 1
half 2
SEA baselines
Δf/f
time (h)
OFCp Activity z-scored Δf/f (STD)
n.s
Wake
NREM
awakening
maintained
retro-AAV-Cre ACC
AAV-DIO-GFP CLA
EEG
EMG
percent time spent in NREM (%)
Saline
CNO
proportion of awakening trials

Figure S1. Supplementary sleep data. (**A**) Distribution of EMG root mean square versus frontal EEG power (ratio between power in high [> 25Hz] versus low [< 5Hz] frequencies) from a representative mouse. Each dot signifies a 4s data epoch. Orange: wake; Green: REM; Blue: NREM. Embedded pie chart (top left) shows average time ratio spent in each state in all mice (*n* = 12). (**B**) Representative traces of frontal (F) and parietal (P) EEG, EMG, and ACCp GCaMP6s signals during a single NREM epoch expanded from (A). Horizontal line represents signal mean over the entire recording. mv – millivolts. (**C**) Average traces of ACCp GCaMP activity around transitions from wake to NREM sleep (time 0) during the first half of recording session (first 6 hours, light gray traces), and the second half of the recording session (last 6 hours, black trace). Note similar dynamics, attesting to the stability of photometry signals across 12-hour recordings. Data is plotted as mean ± s.e.m. (**D**) ACCp activity remained globally stable across hours of recording. Dots depict ACCp baseline (4s) preceding each auditory trial during NREM (*n* = 12 mice). Gaps reflect 30 minute break periods in the recording (Methods). (**E**) OFCp activity by states. Boxes represent the median with 25th and 75th percentiles with whiskers extending to non-outlier extremes. Dots represent individual mice ($n = 6$, $t_{(5)} = -0.66$, $p = 0.54$, *paired t-test*). (**F**) Auditory awakening experiments during NREM sleep. Average trace (± s.e.m) prior to tone-pip. Dashed square represents the time window analyzed. Gray: maintained sleep; Red: awakening. (**G**) OFCp activity preceding the tone. Each dot represents average activity from a single animal ($n = 6$ mice, $t_{(5)} = -0.21$, $p = 0.84$, *paired t-test*). Grey: maintained; Red- awakening. (**H**) Surgical approach for chemogenetic DREADD control group. (**I**) Time spent in NREM sleep in ACCp controls expressing eGFP. Each dot represents a single mouse ($n = 7$, $t_{(6)} = 1.8$, $p = 0.12$, *paired t test*). Grey: CNO injection; Teal- vehicle injection. (**J**) Proportion of awakenings in ACCp controls expressing eGFP ($n = 7$, $t_{(6)} = 1.83$, $p = 0.12$, *paired t test*). Grey: CNO injection; Teal- vehicle injection. Histograms in G, I, J depict the bootstrapped distribution of means with 95% confidence intervals.

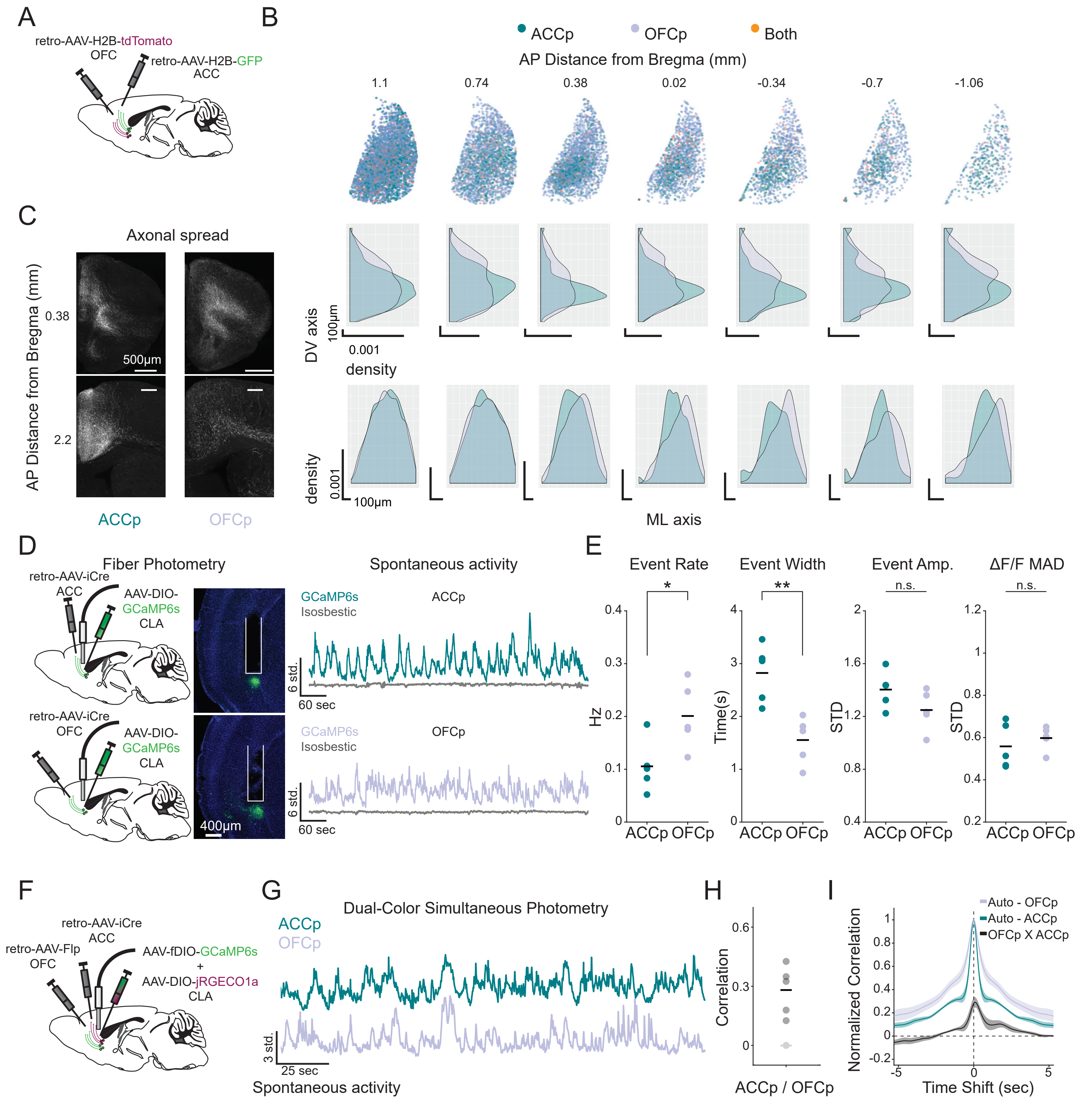

Figure S2. Anatomically distinct claustrum networks projecting to the ACC vs the OFC. Panels **A-C** Relate to differential spatial localization and minimal overlap of the cell bodies of neurons projecting to the ACC vs the OFC. Panels **D-I** relate to differential activity recorded in ACCp and OFCp neurons. (**A**) Scheme for dual-color nuclei-targeted retrograde labelling of claustrum projection neurons. (**B**) Data overlaid from 6 mice showing spread of ACCp and OFCp neurons (top panels) and their respective density distributions along the dorso-ventral (DV; middle panels) or medio-lateral (ML; lower panels) axes of the claustrum. Scale bars represent 100 μm. (**C**) IHC-amplified GFP-labelled ACCp (left) or OFCp (right) axonal projections within ACC (top) and OFC (bottom). Numbers refer to AP distance from bregma. Note the relative enrichment of ACC-projections of ACCp neurons and OFC-projections of OFCp neurons. (**D**) Approach for fiber photometry recordings from ACCp (top) vs OFCp (bottom) claustrum populations. Middle panels depict representative histological expression and optic fiber placement. Right panels depict spontaneous activity in head-restrained mice compared to a co-recorded isosbestic control channel. Movement artifacts were minimal throughout the study. (**E**) Quantification of spontaneous calcium event rate, width (at half maximal prominence), amplitude, and overall median absolute deviation (MAD) of ACCp vs OFCp z-scored ΔF/F $n = 5$ mice in each group. Event rate: $t_{(8)} = -2.687$, $p = 0.0276$, *two-tailed two-sample t-test.* Width: $t_{(8)} = 4.022$, $p = 0.0038$, *two-tailed two-sample t-test.* Amplitude: $t_{(8)} = 1.679$, $p = 0.1317$, *two-tailed two-sample t-test.* MAD: $t_{(8)} = -0.727$, $p = 0.488$, *two-tailed two-*

sample t-test. Spontaneous calcium events were identified with the MATLAB function findpeaks. To avoid multiple identifications of single events or definitions of noise as activity, thresholds of a minimal prominence of 1 standard deviation and a minimum of 2-second event width measured at half prominence were employed. Changing these parameters did not alter the results dramatically. (**F**) Approach for simultaneous recording from ACCp and OFCp neurons using two-color photometry. (**G**) Representative co-recorded spontaneous photometry traces from an ACCp/OFCp mouse. (**H**) Correlation between spontaneous co-activity in ACCp/OFCp mice (n=5). Light gray dot represents the maximal correlation over 1000 iterations of shuffled data per mouse, averaged across mice. (**I**) Average cross-correlation of spontaneous activity in ACCp/OFCp mice (gray, n = 5) in comparison to the auto-correlations of OFCp (purple) and ACCp (cyan).

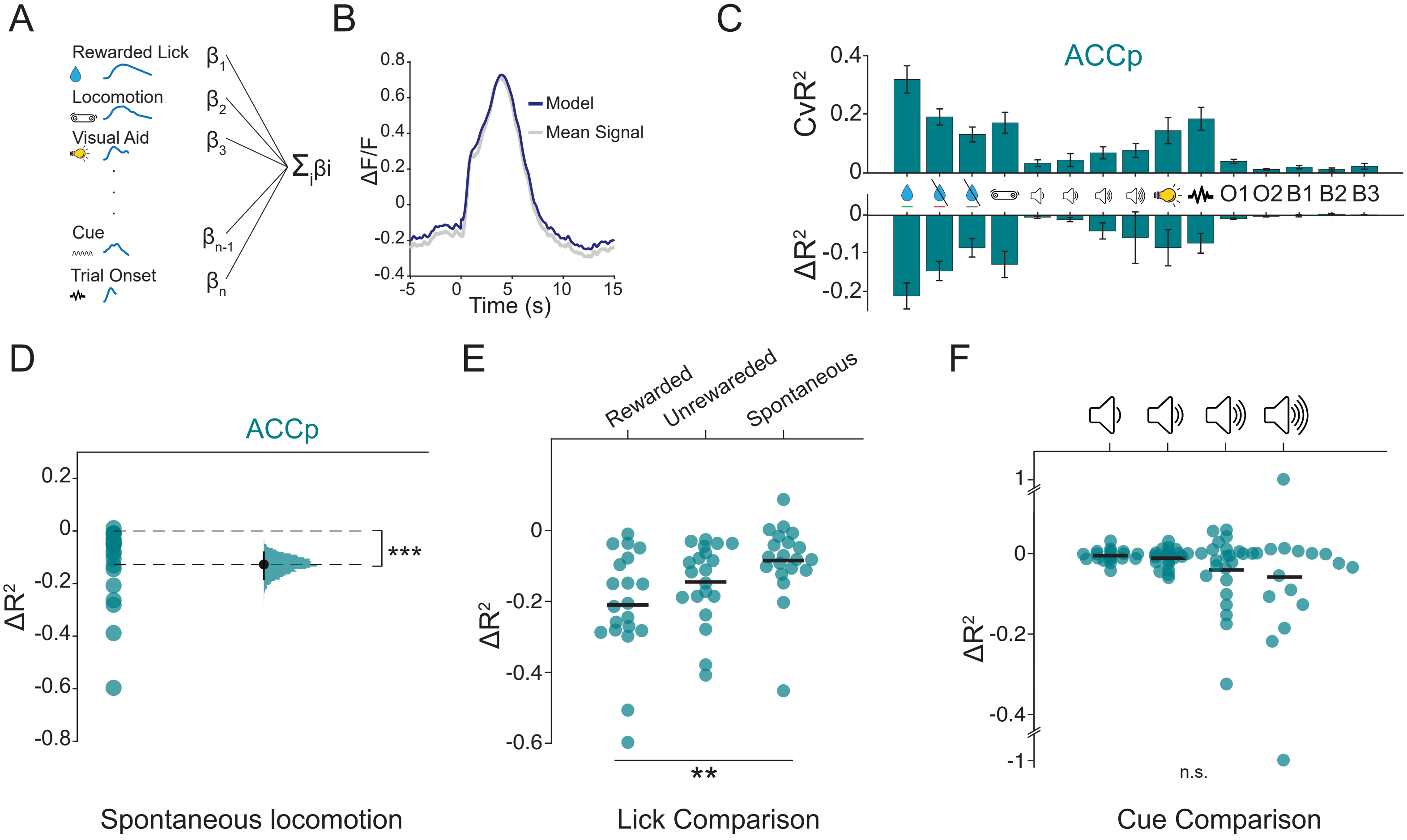

Figure S3. A linear encoding model for quantification of the claustral representation of task parameters in ACCp neurons. (**A-B**) Regressor weights (time-event kernels) were calculated around each event in the task (see also Methods and Supplementary Table 2 for details regarding the model), associating it with its corresponding neural signal. Kernels were linearly summed with equal weights (**A**) to generate a prediction for the average neural signal (**B**). (**C**) Linear encoding model quantification of the contribution of behavioral events to claustrum photometry signal ($n = 20$ ACCp channels). Events, from left to right: rewarded licks (hits); unrewarded licks (impulsive errors); spontaneous licks (outside the trial); locomotion; cue onset (4 intensities); visual aid onset; trial onset (BBN). The relative representation of events in the signal was evaluated by comparing the cross-validated explained variance (CvR^2) obtained by partial models (which are based on each event independently) to the CvR^2 obtained from the full model (constructed from all events). In addition, the unique contribution of each event to the neural signal was measured as the loss of explanatory power caused by omitting that event from the full model (ΔR^2). ΔR^2 was then used as a metric for the contribution of individual events to the claustrum signal. (**D**) Model quantification of the unique contribution of claustrum activity during spontaneous locomotion events in individual mice ($n = 20$ ACCp channels, $p < 0.00001$ *(0/5000 ≥ 0)*, *permutation test on bootstrapped distribution of locomotion unique contribution)*. (**E**) Model quantification of the different unique contribution of rewarded licks (hits); unrewarded licks (impulsive errors) and spontaneous licks (outside the trial) to the ACCp signal ($\underline{n} = 20$ ACCp channels, significant main effect of licking unique contribution, $F_{(2, 58)} = 11.844$, $p = 0.0011$, *ANOVA on linear mixed effects model)*. (**F**) Model quantification of the unique contribution of go-cue stimuli ($\underline{n} = 20$ ACCp channels, no significant main effect of cue intensity unique contribution, $F_{(3, 78)} = 1.785$, $p = 0.198$, *ANOVA on linear mixed effects model)*. Unless noted otherwise, data are mean ± s.e.m. N.S., not significant. See Supplementary Table 3 for further details of the statistical analyses.

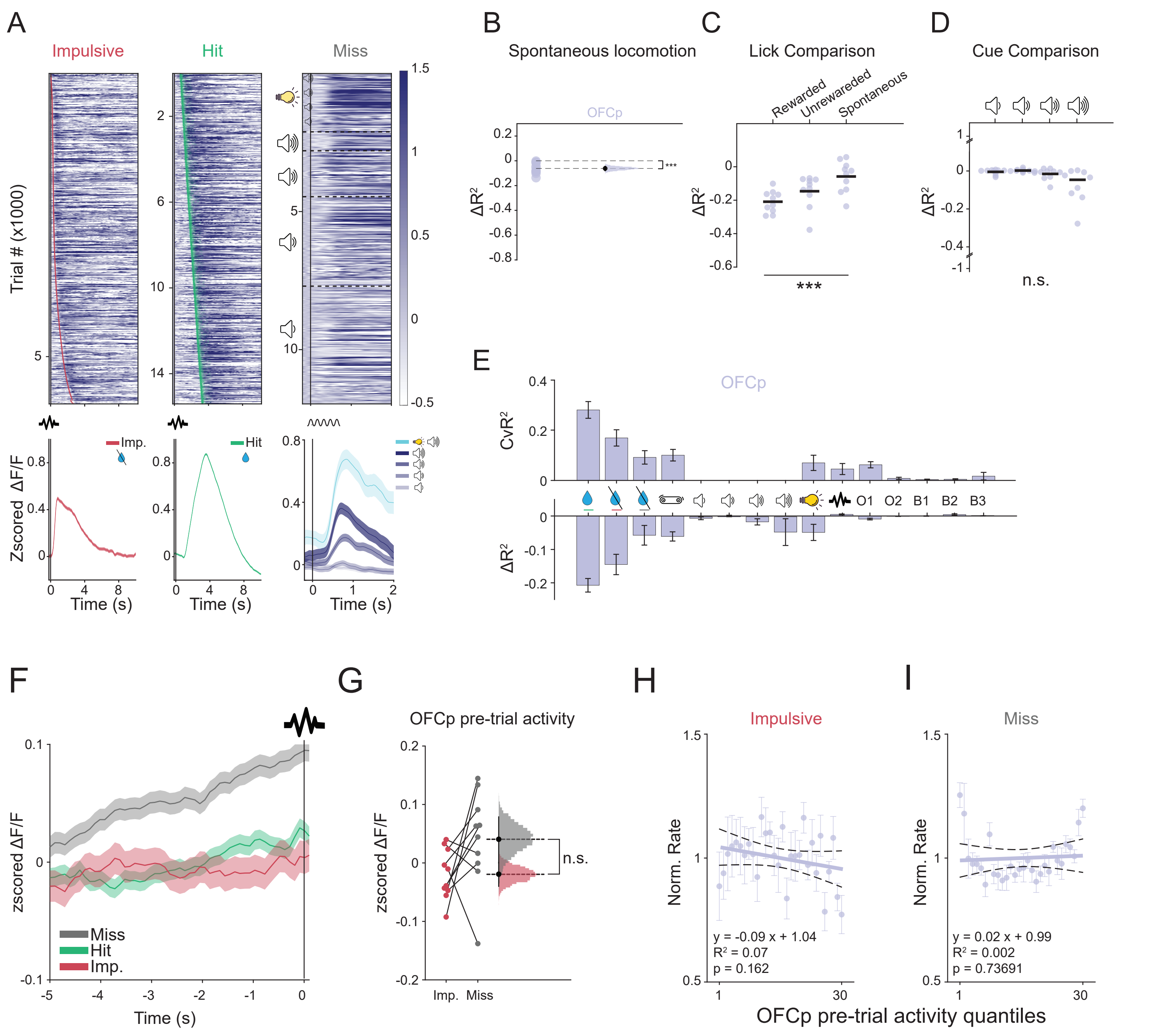

Figure S4. OFCp activity during the ENGAGE task. (**A**) Top: All OFCp trials for impulsive ($n = 5{,}835$), hit ($n = 15{,}409$), and miss ($n = 12{,}815$) trials from $n = 10$ mice, presented as in Figure 2. Red and green ticks in the first two panels indicate the first impulsive or correct lick within the trial, respectively. Bottom: mean activity traces in impulsive (left) hit (middle) and miss trials (right, separated by cue intensity). Shaded area represents s.e.m. The vertical black line indicates trial onset (for impulsive & hit trials) or cue (for miss trials). (**B**) Linear encoding model quantification of the contribution of spontaneous locomotion to claustrum photometry signal ($n = 20$ ACCp channels, $p < 0.00001$ *(0/5000 ≥ 0), permutation test on bootstrapped distribution of locomotion unique contribution)*. (**C**) Model quantification of the different unique contribution of rewarded licks (hits); unrewarded licks (impulsive errors) and spontaneous licks (outside the trial) to OFCp activity in individual mice ($n = 10$ OFCp channels, significant main effect of licking unique contribution, $F_{(2, 58)} = 16.98$, $p = 0.0003$, *ANOVA on linear mixed effects model)*. (**D**) Model quantification of the unique contribution of go-cue stimuli to OFCp activity ($n = 10$ OFCp channels, no significant main effect of cue intensity unique contribution, $F_{(3, 38)} = 2.5585$, $p = 0.118$, *ANOVA on linear mixed effects model)*. (**E**) Linear encoding model quantification of the contribution of behavioral events to claustrum photometry signal ($n = 10$ OFCp channels). As in Figure S3C. (**F**) Pre-trial OFCp activity averaged over mice ($n = 20$) aligned to trial onset and separated by trial outcome. (**G**) OFCp pre-trial baseline activity in impulsive trials (red) and miss trials (gray), depicted as individual mice and bootstrapped distribution of means with 95% confidence intervals ($n = 10$ OFCp *mice*, $t_{(9)} = 1.766$, $p = 0.113$, *paired t-test*). (**H-I**) Normalized impulsive (**H**) or miss (**I**) error rate, as a function of pre-trial activity quantiles for OFCp. (**H** $n = 10$, $p = 0.162$; $R^2 = 0.07$. *simple linear regression.* **I** $n = 10$, $p = 0.737$; $R^2 < 0.01$, *simple linear regression)*. Thick lines represent linear fit, dotted lines represent 95% confidence intervals. Unless noted otherwise, data are mean ± s.e.m.

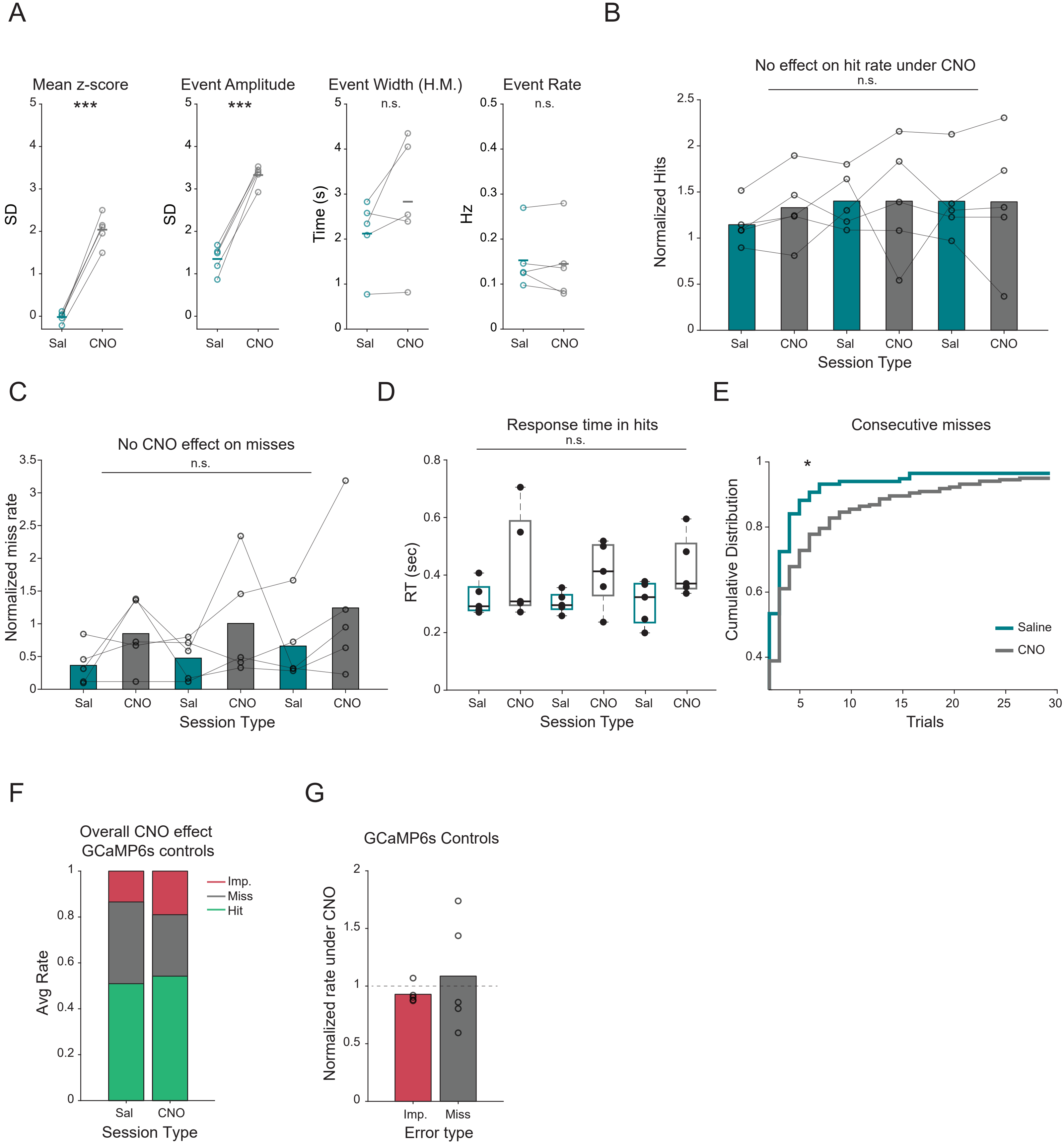

Figure S5. Supplementary data relating to figure 3. (**A**) Analyses of the impact of CNO (10mg/kg i.p) injections on periodicity and magnitude of ACCp signals as compared to saline injections. Median absolute deviation (MAD) of z-scored ΔF/F, event amplitude, width (at half maximal prominence), and rate ($n = 5$ mice; MAD: $t_{(8)} = -0.702$, $p = 0.521$, *two-tailed paired t-test*; Amplitude: $t_{(4)} = 14.744$, $p < 0.0001$, *two-tailed paired t-test*; Width: $t_{(4)} = 1.754$, $p = 0.154$, *two-tailed paired t-test*; Event rate: $t_{(4)} = 14.973$, $p < 0.0001$, *two-tailed paired t-test*). (**B**) Hit rates in interleaved daily sessions of saline vs CNO ($\underline{n} = 5$, $F_{(1, 26)} = 0.48$, $p = 0.495$; $F_{(4, 26)} = 1.686$, $p = 0.206$, No significant main effects of treatment and repetition, respectively, and no significant interaction $F_{(4, 26)} = 0.476$, $p = 0.497$, *ANOVA on linear mixed effects model*). Connected circles indicate individual mice. Hit rates are normalized to the average rate over 3 prior days of saline habituation. (**C**) Miss rates in interleaved daily sessions of saline vs CNO ($\underline{n} = 5$, $F_{(1, 26)} = 2.39$, $p = 0.134$; $F_{(4, 26)} = 0.83$, $p = 0.371$. No significant main effects of treatment and repetition, respectively, and no significant interaction $F_{(4, 26)} = 0.25$, $p = 0.623$,

ANOVA on linear mixed effects model). Connected circles indicate individual mice. Error rates are normalized to the average rate over 3 prior days of saline habituation. (**D**) Response times in hit trials throughout the experiment were not affected by CNO ($\underline{n}$ = 5, $F_{(1, 26)}$ = 1.53, p = 0256; $F_{(4, 26)}$ = 0.006, p = 0.806. No significant main effects of treatment and repetition, respectively, and no significant interaction $F_{(4, 26)}$ = 0.034, p = 0.855, *ANOVA on linear mixed effects model*). Boxes represent group median and 25th and 75th percentiles with whiskers extending to non-outlier extremes. (**E**) Cumulative probability distribution of consecutive miss trials in saline (turquoise) and CNO (gray) sessions (n = 5; D = 0.132, p =0.013, *Two-sample Kolmogorov-Smirnov test*). (**F**) Overall average trial outcomes for control mice (n = 5). (**G**) No significant changes in error rates (normalized to two baseline saline sessions) following CNO (10mg/kg i.p) in GCaMP6s controls ($\underline{n}$ = 5, $t_{(4)}$ = -1.969, p = 0.1204, $t_{(4)}$ = 0.404, p = 0.707, *student's t-test* for impulsive errors and misses, respectively). Unless noted otherwise, data are mean ± s.e.m.

Supplementary Tables

Supplementary Table 1: Surgeries				
Experiment	Constructs injected	Number of mice	Injection site	Figure
ACCp single channel claustrum + EEG photometry recordings	retroAAV-Cre (ACC) (ELSC virus vector core) AAV-DIO-GCaMP6s (CLA) (Addgene 100842)	6	ACC: LM ±0.4; RC: 1.1; DV -1.75 CLA: LM ±3.3; RC: 0; DV: -4.15	1, S1
OFCp single channel claustrum + EEG photometry recordings	retroAAV-Cre (OFC) AAV-DIO-GCaMP6s (CLA)	7	OFC: LM ±1; RC: +2.55; DV: -2.3; CLA: LM ±3.3; RC: 0; DV: -4.15	S1
Chemogenetic activation of ACCp neurons during sleep	retroAAV-Cre (ACC) AVV-DIO-GCaMP6s (CLA) AAV-DIO-hM3Dq-mCherry	6 (2 ACC and 2 CLA injections in each hemisphere)	ACC: LM ±0.4; RC: 1.1 & 0.5; DV -1.75 CLA: LM ±3.3 & ±2.85; RC: 0 & +1; DV: -4.15 & -3.75	1, S1
Anatomical mapping of ACCp and OFCp populations	retroAAV-H2B-GFP (ELSC virus vector core) retroAAV-H2B-tdTomato (ELSC virus vector core)	6 ACC/OFC	OFC: LM ±1; RC: +2.55; DV: -2.3; ACC: LM ±0.4; RC: 1.1; DV -1.75	S2
ACCp single channel claustrum photometry recordings	retroAAV-Cre (ACC) AAV-DIO-GCaMP6s (CLA)	5	ACC: LM ±0.4; RC: 1.1; DV -1.75 CLA: LM ±3.3; RC: 0; DV: -4.15	2, 4, S2, S3, S6
OFCp single channel claustrum photometry recordings	retroAAV-Cre (OFC) AAV-DIO-GCaMP6s (CLA)	5	OFC: LM ±1; RC: +2.55; DV: -2.3; CLA: LM ±3.3; RC: 0; DV: -4.15	2, 4, S2, S4, S6
Dual channel claustrum recordings	retroAAV-Cre (ACC) retroAAV-flp (OFC) (ELSC virus vector core) AAV-DIO-jRGECO1a (CLA) (ELSC virus vector core) AAV-fDIO-GCaMP6s (CLA) (ELSC virus vector core)	4	OFC: LM ±1; RC: +2.55; DV: -2.3; ACC: LM ±0.4; RC: 1.1; DV -1.75 CLA: LM ±3.3; RC: 0; DV: -4.15	2, 4, S2, S3, S4, S6
Dual channel claustrum recordings	retroAAV-Cre (OFC) retroAAV-flp (ACC) AAV-DIO-jRGECO1a (CLA) AAV-fDIO- GCaMP6s (CLA)	1	OFC: LM ±1; RC: +2.55; DV: -2.3; ACC: LM ±0.4; RC: 1.1; DV -1.75 CLA: LM ±3.3; RC: 0; DV: -4.15;	2, 4, S2, S3, S4, S6
Dual channel claustrum recordings	retroAAV-Cre (ACC) retroAAV-flp (AUD) AAV-DIO-jRGECO1a (CLA) AAV-fDIO-GCaMP6s (AUD) (data not shown)	1	AUD: LM ±4.1; RC: -2.8; DV: -2.4; ACC: LM ±0.4; RC: 1.1; DV -1.75 CLA: LM ±3.3; RC: 0; DV: -4.15	2, 4, S2, S3, S6
Dual channel claustrum recordings	retroAAV-Cre (AUD) retroAAV-flp (ACC) AAV-DIO-jRGECO1a (CLA) AAV-fDIO-GCaMP6s (AUD) (data not shown)	2: In one mouse two hemispheres were recorded in separate experiments for 3 data sets in total	AUD: LM ±4.1; RC: -2.8; DV: -2.4; ACC: LM ±0.4; RC: 1.1; DV -1.75 CLA: LM ±3.3; RC: 0; DV: -4.15	2, 4, S2, S3, S6
Dual channel claustrum recordings	retroAAV-Cre (ACC) AAV-axon-GCaMP6s (Addgene 112005) (ACC) (data not shown) AVV-DIO-jRGECO1a (CLA)	3	ACC: LM ±0.4; RC: 1.1; DV -1.75 CLA: LM ±3.3; RC: 0; DV: -4.15	2, 4, S2, S3, S6
Dual channel claustrum recordings	retroAAV-Cre (ACC) AAV-axon-GCaMP6s (AUD) (data not shown) AVV-DIO-jRGECO1a (CLA)	3	AUD: LM ±4.1; RC: -2.8; DV: -2.4; CLA: LM ±3.3; RC: 0; DV: -4.15	2, 4, S2, S3, S6

Chemogenetic activation of ACCp neurons	retroAAV-Cre (ACC)<br>AVV-DIO-GCaMP6s (CLA)<br>AAV-DIO-hM3Dq-mCherry	5 (2 ACC and 2 CLA injections in each hemisphere)	ACC: LM ±0.4; RC: 1.1 & 0.5; DV -1.75<br>CLA: LM ±3.3 & ±2.85; RC: 0 & +1; DV: -4.15 & -3.75	3, S5
Additional GCaMP6s controls for chemogenetic experiments	retroAAV-Cre (ACC)<br>AAV-DIO-GCaMP6s (CLA)	3	ACC: LM ±0.4; RC: 1.1; DV -1.75<br>CLA: LM ±3.3; RC: 0; DV: -4.15	S5

Supplementary Table 2: Epochs for time-event kernels in the linear encoding model

Variable name	Description	Time window	Selected from event(s):	ACCp CVR^2 and ΔR^2 (mean±sem)	OFCp CVR^2 and ΔR^2 (mean±sem)
Rewarded licks	Lick events from hit trials	(-0.5,5)	First lick in hit trials	0.316 ± 0.046<br><br>-0.21 ± 0.034	0.275 ± 0.033<br><br>-0.21 ± 0.02
Unrewarded licks	Lick events from impulsive error trials	(-0.5,5)	First lick in impulsive error trials	0.189 ± 0.027<br><br>-0.145 ± 0.025	0.166 ± 0.032<br><br>-0.146 ± 0.03
Spontaneous licks	Lick events not associated with the task	(-0.5,5)	Licks at times (-5,0) or (5,15) relative to trial onset	0.123 ± 0.025<br><br>-0.085 ± 0.024	0.089 ± 0.026<br><br>-0.058 ± 0.03
Spontaneous runs	Locomotion events outside the task	(-0.5,5)	Runs at times (-5,0) or (5,15) relative to trial onset	0.169 ± 0.035<br><br>-0.128 ± 0.034	0.099 ± 0.023<br><br>-0.062 ± 0.014
Auditory cue (4 attenuations)	Auditory 'Go' cue	(0,3)	Only from miss trials without visual aid	0.033 ± 0.011<br>0.044 ± 0.022<br>0.068 ± 0.02<br>0.076 ± 0.023<br><br>-0.005 ± 0.003<br>-0.011 ± 0.005<br>-0.041 ± 0.021<br>-0.058 ± 0.067	0.006 ± 0.003<br>0.017 ± 0.007<br>0.009 ± 0.003<br>0.077 ± 0.032<br><br>-0.005 ± 0.043<br>0.002 ± 0.002<br>-0.016 ± 0.009<br>-0.04 ± 0.0403
Visual stimulation	Visual aid LED stimulus	(0,3)	Only from miss trials with visual aid (compounded on auditory cues)	0.143 ± 0.044<br><br>-0.084 ± 0.047	0.069 ± 0.029<br><br>-0.049 ± 0.024
BBN	Trial onset	(0,1)	Miss trials and trials w/ first lick at >1 sec from trial onset	0.183 ± 0.038<br><br>-0.073 ± 0.026	0.045 ± 0.021<br><br>0.005 ± 0.001
Decay after correct trial	Activity following hit	(-10,0)	last 10sec of trial	0.039 ± 0.007<br><br>-0.009 ± 0.002	0.06 ± 0.012<br><br>-0.009 ± 0.002
Decay after incorrect trial	Activity following impulsive/miss trials	(-10,0)	last 10sec of trial	0.008 ± 0.0043<br><br>-0.003 ± 0.001	0.008 ± 0.004<br><br>-0.0008 ± 0.0004
Pre-trial following correct trials	Pre-trial activity following hit	(0,5)	Based on previous trial outcome	0.019 ± 0.006<br><br>-0.0013 ± 0.003	0.003 ± 0.002<br><br>-0.0001 ± 0.0006
Pre-trial following miss trials	Pre-trial activity following miss	(0,5)	Based on previous trial outcome	0.022 ± 0.001<br><br>0.0008 ± 0.001	0.017 ± 0.015<br><br>0.0013 ± 0.001
Pre-trial following impulsive trials	Pre-trial activity following impulse errors	(0,5)	Based on previous trial outcome	0.012 ± 0.005<br><br>0.003 ± 0.0015	0.004 ± 0.002<br><br>0.004 ± 0.0023
