# Supplementary material for: Claustrum neurons projecting to the anterior cingulate restrict engagement during sleep and behavior
Source: Nat Commun. 2024 Jun 26;15:5415. doi: 10.1038/s41467-024-48829-6 (PMC11208603; doi:10.1038/s41467-024-48829-6)
Supplement: Supplementary file 3 — Reporting Summary [file 41467_2024_48829_MOESM3_ESM.pdf]

nature portfolio

Corresponding author(s): Ami Citri

Last updated by author(s): April 16 2024

Reporting Summary

Nature Portfolio wishes to improve the reproducibility of the work that we publish. This form provides structure for consistency and transparency in reporting. For further information on Nature Portfolio policies, see our Editorial Policies and the Editorial Policy Checklist.

Statistics

For all statistical analyses, confirm that the following items are present in the figure legend, table legend, main text, or Methods section.

n/a	Confirmed	
☐	☒	The exact sample size (*n*) for each experimental group/condition, given as a discrete number and unit of measurement
☐	☒	A statement on whether measurements were taken from distinct samples or whether the same sample was measured repeatedly
☐	☒	The statistical test(s) used AND whether they are one- or two-sided<br>*Only common tests should be described solely by name; describe more complex techniques in the Methods section.*
☐	☒	A description of all covariates tested
☐	☒	A description of any assumptions or corrections, such as tests of normality and adjustment for multiple comparisons
☐	☒	A full description of the statistical parameters including central tendency (e.g. means) or other basic estimates (e.g. regression coefficient) AND variation (e.g. standard deviation) or associated estimates of uncertainty (e.g. confidence intervals)
☐	☒	For null hypothesis testing, the test statistic (e.g. *F*, *t*, *r*) with confidence intervals, effect sizes, degrees of freedom and *P* value noted<br>*Give P values as exact values whenever suitable.*
☒	☐	For Bayesian analysis, information on the choice of priors and Markov chain Monte Carlo settings
☒	☐	For hierarchical and complex designs, identification of the appropriate level for tests and full reporting of outcomes
☒	☐	Estimates of effect sizes (e.g. Cohen's *d*, Pearson's *r*), indicating how they were calculated

Our web collection on statistics for biologists contains articles on many of the points above.

Software and code

Policy information about availability of computer code

Data collection	Synapse (Tucker Davis Technologies), MATLAB (Mathworks); custom code available at: https://github.com/Citrilab/Atlan_et_al_2024
Data analysis	MATLAB (Mathworks), custom code available at: https://github.com/Citrilab/Atlan_et_al_2024

For manuscripts utilizing custom algorithms or software that are central to the research but not yet described in published literature, software must be made available to editors and reviewers. We strongly encourage code deposition in a community repository (e.g. GitHub). See the Nature Portfolio guidelines for submitting code & software for further information.

Data

Policy information about availability of data

All manuscripts must include a data availability statement. This statement should provide the following information, where applicable:

- Accession codes, unique identifiers, or web links for publicly available datasets
- A description of any restrictions on data availability
- For clinical datasets or third party data, please ensure that the statement adheres to our policy

Full data (XXXXX) and code have been uploaded to public repositories

Research involving human participants, their data, or biological material

Policy information about studies with human participants or human data. See also policy information about sex, gender (identity/presentation), and sexual orientation and race, ethnicity and racism.

Reporting on sex and gender	n/a
Reporting on race, ethnicity, or other socially relevant groupings	n/a
Population characteristics	n/a
Recruitment	n.a
Ethics oversight	n/a

Note that full information on the approval of the study protocol must also be provided in the manuscript.

Field-specific reporting

Please select the one below that is the best fit for your research. If you are not sure, read the appropriate sections before making your selection.

☒ Life sciences ☐ Behavioural & social sciences ☐ Ecological, evolutionary & environmental sciences

For a reference copy of the document with all sections, see nature.com/documents/nr-reporting-summary-flat.pdf

Life sciences study design

All studies must disclose on these points even when the disclosure is negative.

Sample size	No statistical methods were used to pre-determine sample sizes. Sample sizes in this paper were chosen to be similar to those used in previous publications in the field.
Data exclusions	No data were excluded from this study.
Replication	All findings were replicated across multiple independent samples and at least two independent experiments. The number of samples for each experiment is detailed in the manuscript.
Randomization	Only one genotype of male mice (C57BL/6JOLAHSD) was used throughout the study. Animals were randomly selected for injection target (ACCp/OFCp), and all mixed linear models include mouse identity as an independent covariable.
Blinding	Data collection was performed a largely blind fashion, as cohorts were based on mixed control and experimental animals, which were co-housed, co-trained and recorded over multiple sessions under identical conditions, with no relation to grouping. Analyses were blinded as they were performed using code that ran on all experimental and control animals simultaneously.

Reporting for specific materials, systems and methods

We require information from authors about some types of materials, experimental systems and methods used in many studies. Here, indicate whether each material, system or method listed is relevant to your study. If you are not sure if a list item applies to your research, read the appropriate section before selecting a response.

Materials & experimental systems

n/a	Involved in the study	
☐	☒	Antibodies
☒	☐	Eukaryotic cell lines
☒	☐	Palaeontology and archaeology
☐	☒	Animals and other organisms
☒	☐	Clinical data
☒	☐	Dual use research of concern
☒	☐	Plants

Methods

n/a	Involved in the study	
☒	☐	ChIP-seq
☒	☐	Flow cytometry
☒	☐	MRI-based neuroimaging

Antibodies

Antibodies used	rabbit anti-GFP, Life Technologies, Bethesda, MD; catalog No. A-6455; final dilution to 1:500 in 3% normal horse serum
Validation	Validation statements from the supplier websites:<br>For rabbit anti-GFP:<br>This antibody was verified by Relative expression to ensure that the antibody binds to the antigen stated.

Animals and other research organisms

Policy information about studies involving animals; ARRIVE guidelines recommended for reporting animal research, and Sex and Gender in Research

Laboratory animals	All mice described in this study were male C57BL/6JOLAHSD obtained from Harlan Laboratories, Jerusalem, Israel. Mice were 6-8 weeks old at the time of surgery and trained and tested for a period of several weeks to several months (in the case of mice transported to TAU).
Wild animals	This study did not involve wild animals.
Reporting on sex	All mice used in this study were male.
Field-collected samples	This study did not involve field-collected samples.
Ethics oversight	All experimental procedures, handling, surgeries, and care of laboratory animals used in this study were approved by the Hebrew University Institutional Animal Care and Use Committee (NS-19-15584-3; NS-19-15788-3) and by the Institutional Animal Care and Use Committee (IACUC) of Tel Aviv University (approval 01-19-078).

Note that full information on the approval of the study protocol must also be provided in the manuscript.

Plants

Seed stocks	n/a
Novel plant genotypes	n/a
Authentication	n/a
